# Mouse Antibody of IgM Class is Prone to Non-Enzymatic Cleavage between CH1 and CH2 Domains

**DOI:** 10.1038/s41598-017-19003-4

**Published:** 2018-01-11

**Authors:** Tomasz Klaus, Krystyna Stalińska, Dominik Czaplicki, Paweł Mak, Bozena Skupien-Rabian, Sylwia Kedracka-Krok, Karolina Wiatrowska, Monika Bzowska, Monika Machula, Joanna Bereta

**Affiliations:** 1 Malopolska Centre of Biotechnology, Jagiellonian University in Kraków, Gronostajowa 7A, 30-387 Kraków, Poland; 2 Department of Cell Biochemistry, Jagiellonian University in Kraków, Gronostajowa 7, 30-387 Kraków, Poland; 3 Department of Analytical Biochemistry, Jagiellonian University in Kraków, Gronostajowa 7, 30-387 Kraków, Poland; 4 Department of Physical Biochemistry, Faculty of Biochemistry, Biophysics and Biotechnology, Jagiellonian University in Kraków, Gronostajowa 7, 30-387 Kraków, Poland

## Abstract

IgM is a multivalent antibody which evolved as a first line defense of adaptive immunity. It consists of heavy and light chains assembled into a complex oligomer. In mouse serum there are two forms of IgM, a full-length and a truncated one. The latter contains μ’ chain, which lacks a variable region. Although μ’ chain was discovered many years ago, its origin has not yet been elucidated. Our results indicate that μ’ chain is generated from a full-length heavy chain by non-enzymatic cleavage of the protein backbone. The cleavage occurred specifically after Asn209 and is prevented by mutating this residue into any other amino acid. The process requires the presence of other proteins, preferentially with an acidic isoelectric point, and is facilitated by neutral or alkaline pH. This unique characteristic of the investigated phenomenon distinguishes it from other, already described, Asn-dependent protein reactions. A single IgM molecule is able to bind up to 12 epitopes via its antigen binding fragments (Fabs). The cleavage at Asn209 generates truncated IgM molecules and free Fabs, resulting in a reduced IgM valence and probably affecting IgM functionality *in vivo*.

## Introduction

IgMs are the first antibodies produced by B-cells during immune response. They are very large proteins with a molecular mass (Mw) of about 1 MDa. A complete IgM molecule consists of three types of polypeptides: heavy chains, light chains and J-chain. N-terminal parts of each heavy and light chains consist of unique variable domains that form an antigen binding site. The remaining parts of the heavy and light chains are constant regions shared by all IgMs. The heavy chain of IgM has four constant domains, called CH1-CH4, and the light chain has one, CL. Two heavy chains and two light chains associate into a monomeric IgM. CH4 domain has an intrinsic ability to oligomerize^1^ and induces formation of IgM penta- or hexamers. IgM oligomers are covalently linked via disulfide bonds and their structures resemble snowflakes. J-chain is a 15 kDa polypeptide crucial for IgM secretion into mucosa^2^.

Mouse IgMs are widely used in research and diagnostics, especially in blood typing. Moreover, IgMs, including those of mouse origin^3,4^, are considered as promising therapeutic agents with strong cytotoxic potential. The framework of IgM may be very useful in therapies due to its high avidity and fast pharmacokinetics. However, the clinical application of IgMs is challenging because of many problems with production and downstream processing^5,6^.

High Mw, heavy glycosylation, dozens of disulfide bonds and a complex oligomeric structure make IgMs very difficult to express, purify and formulate. IgMs activity is rapidly lost through aggregation that is predominantly disulfide driven^7^. In the case of mouse IgMs we observed additional phenomenon adversely affecting their activity: the heavy chain N-terminus trimming, which yields 55 kDa μ’ chain^7^.

The first description of μ’ chain was given by Marks and Bosma in 1985^8^. Although the authors proved that μ’ chain is a fragment of a heavy chain lacking a variable region, the origin and exact sequence of μ’ chain were unknown.

To date, apart from the mouse μ’ chain, at least four examples of a truncated IgM heavy chain have been reported. In two of them the shortened variants are produced via alternative splicing. Each domain of the IgM heavy chain is encoded by a separate exon. In human B-cells the exon coding for a variable domain may be omitted during pro-mRNA splicing^9^ resulting in the heavy chains that lack this domain. Alternative splicing was also postulated as a mechanism of μ’ chain production in mouse cells^8^ but this hypothesis failed to be confirmed^9^.

Another example is an IgM variant produced by bony fish (teleosts). The variant is shortened on C-terminus; it lacks CH4 domain and is a part of the B-cell receptor (BCR) on fish lymphocytes. Bony fish have an exceptional IgM pre-mRNA splicing pattern unique among vertebrates^10^.

The other two examples concern the generation of a shortened mouse IgM heavy chain during B-cell differentiation. This kind of molecule may result from a defective genomic rearrangement^11^. Genes coding for immunoglobulins are formed during B-cell development in a multi-step process called genomic rearrangement. The rearrangement may be stopped by an incorrect assembly, which may lead to production of a heavy chain shortened on N-terminus.

Also, IgM heavy chain that does not associate with a light chain may be structurally labile and prone to proteolytic truncation^12^. The proteolysis was observed intracellularly at an early stage of B-cell development, before the cell started to produce a light chain. Mw of the heavy chain fragment generated by the action of a putative intracellular protease was approximately 48 kDa, which is different from the estimated Mw of μ’ chain (55 kDa).

We have previously reported that the quantity of μ’ chain increases during storage of IgM-based blood typing reagents^7^. Our results indicated that μ’ chain is generated extracellularly from full-length IgM in the presence of serum or in the presence of bovine serum albumin (BSA) preparation, which, apart from albumin, contains numerous other proteins. We have observed that the accumulation of μ’ chain adversely affected the activity of the IgM reagents. Substantial amount of μ’ chain was detected also in normal mouse serum^7,8^, but its influence on immune response and IgM activity *in vivo* is unknown.

Our research aimed to identify the factors involved in μ’ chain generation. We scrutinized the biochemical properties of μ’ chain and determined its sequence. The specific cleavage site, Asn209 in CH1 domain, suggested an enzyme-mediated process. However, our thorough search for the enzyme responsible for IgM truncation led us to the conclusion that the generation of μ’ chain is a non-enzymatic process. In the course of our work we also developed a set of IgM muteins that are resistant to truncation.

## Results

### Mouse μ’ chain is a heavy chain fragment lacking a variable region and a large part of CH1 domain

**Figure 1:**
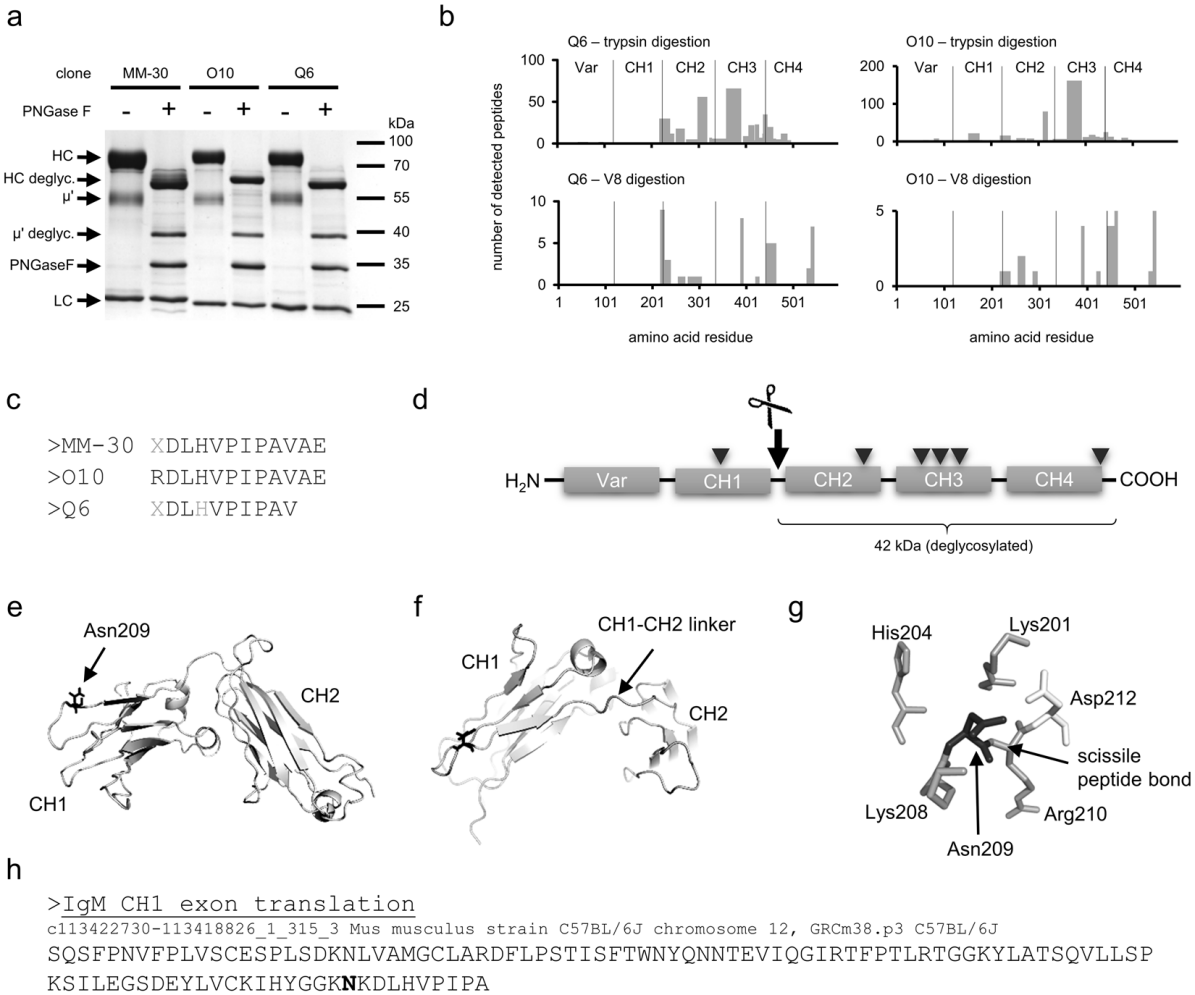
Mouse μ’ chain is generated by specific cleavage after Asn209 in IgM heavy chain constant region. (**a**) Estimation of μ’ chain molecular mass. Three different IgMs were deglycosylated using PNGase F. Glycosylated μ‘ chain is a 55 kDa protein. It consists of a ∼40 kDa polypeptide and five N-glycans, which total Mw is about 15 kDa. The gel was stained using silver nitrate. deglyc. – deglycosylated; HC – heavy chain; LC – light chain. (**b**) MS analysis of μ’ chains derived from O10 and Q6 IgMs. The charts present sequence coverage and numbers of detected peptides obtained from μ’ chain by trypsin- or V8 digestion. (**c**) N-terminal sequences of μ’ chains derived from three different IgMs determined by Edman degradation. Grey letters indicate uncertain residues. (**d**) Domains of full-length IgM heavy chain. Arrowheads indicate N-glycosylation sites. The site of cleavage resulting in μ’ chain is shown with an arrow. (**e**) Model of CH1 and CH2 domains of mouse IgM. Asn209 is in a solvent accessible loop. The model was generated using I-TASSER server^47,48^ and visualized in PyMOL (Schrödinger). (**f**) Asn209 precedes the CH1-CH2 linker. (**g**) Asn209 is surrounded by charged amino acids. (**h**) IgM heavy chain cleavage occurs within CH1 domain after Asn209 (marked in bold). There is a polymorphism in amino acid residue 209 between mouse strains C57 (Lys209) and Balb/c (Arg209).

We began our research from biochemical analyses of μ’ chain. Firstly, we removed potential N-glycans from three different IgMs: MM-30, O10, and Q6, using PNGase F. The experiment revealed that μ’ chain is a glycosylated protein consisting of a polypeptide with Mw of about 40 kDa and several N-glycans, which total Mw is approximately 15 kDa (Fig. 1a). Assuming that Mw of a mammalian N-glycan is approximately 3 kDa, we estimated that 5 N-glycans are attached to the μ’ chain. Mw of μ’ chain polypeptide corresponds to three domains of IgM heavy chain. Considering that the full-length IgM heavy chain consists of five similar-size domains and six N-glycans attached to constant domains, the analysis suggested that μ’ chain may arise in two different ways: (*i*) the molecule is devoid of both N- and C-termini; the variable and CH4 domains are cut off from the full-length heavy chain or (*ii*) truncation occurs only on N-terminus and variable and CH1 domains are removed.

To verify which domains are present in μ’ chain we performed polypeptide sequencing using mass spectrometry (MS). The μ’ chains derived from O10 and Q6 antibodies were fragmented with trypsin or V8 protease. Figure 1b presents coverage and the numbers of measured peptides derived from the full-length heavy chain. Peptides originated from the CH2 and CH3 domains are prevalent independently on the fragmentation method. In contrast to V8 digestion results, CH4-derived peptides were rarely detected after trypsin cleavage. CH4 domain has only few unevenly distributed trypsin cleavage sites. Long peptides are difficult to be efficiently sequenced with MS and probably for this reason we did not observe CH4-derived peptides within tryptic fragments. For both enzymes, the least frequently represented peptides came from variable and CH1 domains. These results indicated that μ’ chain is formed from a full-length heavy chain by cutting off the variable and CH1 domains. Tiny amounts of peptides derived from CH1 domain in O10 samples may result from high sensitivity of MS and incomplete separation of full-length and truncated heavy chains during SDS-PAGE preceding the analysis.

Edman sequencing of μ’ chain N-termini of three different IgMs confirmed the results of MS. (Fig. 1c). The obtained sequences were exactly the same for the three analyzed antibodies and their alignment with the sequence of mouse IgM constant region (UniProtKB record P01872; IGHM_MOUSE) showed that μ’ chain arises by cleavage after Asn209 (EU numeration^13^; Asn209 corresponds to Asn96 in the UniProtKB record). The scissile peptide bond between Asn209 and Arg210 is localized within a solvent accessible loop (Fig. 1e) and precedes the linker between CH1 and CH2 domains (Fig. 1f). The cleavage site is surrounded by basic residues (Fig. 1g).

It has been shown that the truncated human IgM heavy chain is produced by alternative splicing^9^. To exclude the possibility that we identified an alternatively spliced variant of the mouse IgM heavy chain we translated *in silico* the exon coding for CH1 domain (Fig. 1h). The putative cleavage site is localized 9 residues before the end of CH1 domain determined on the basis of exon splicing.

In this part of our research we discovered that μ’ chain is a fragment of IgM heavy chain lacking a variable region and a large part of CH1 domain. The μ’ chain is generated post-translationally by precise cleavage just before the linker between CH1 and CH2 domains.

### Mutating Asn209 prevents cleavage of mouse IgM heavy chain

**Figure 2:**
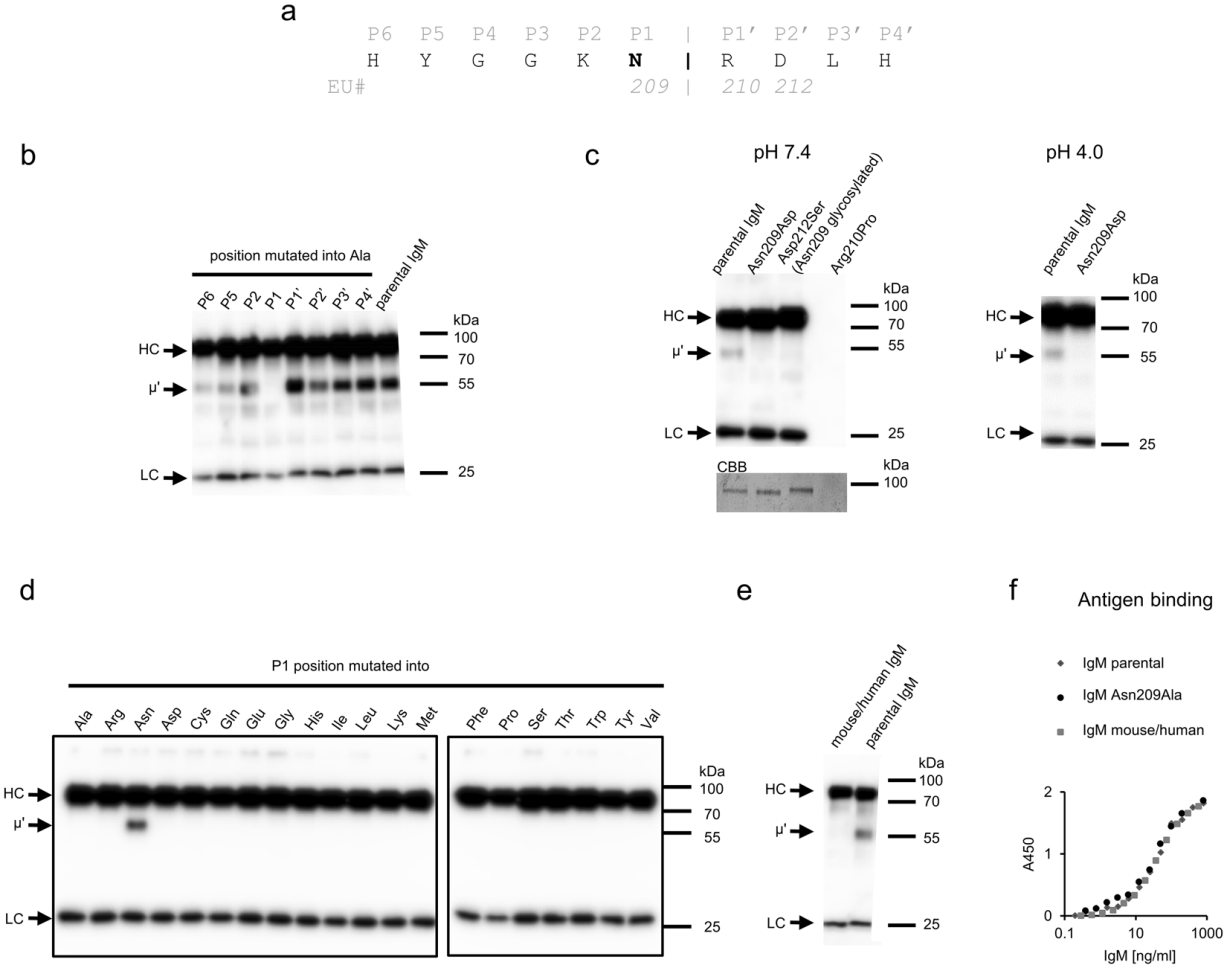
Asn209 in the constant region of mouse IgM heavy chain is crucial for extracellular cleavage of the antibody. (**a**) The scheme presents residues from the loop containing Asn209 that were subjected to alanine screening. Glycine residues at P3 and P4 were not mutated. (**b**–**e**) Stability of mutated IgMs in the presence of serum. μ’ chain was detected using western blotting. The samples were probed with anti-mouse-IgMκ antibody. (**b**) Stability of muteins generated using alanine screening. (**c**) Stability of Asn209Asp and Asp212Ser (N-glycosylated Asn209) muteins. Asn209Asp mutein was incubated at neutral or acidic pH. Despite many efforts we were not able to produce Arg210Pro mutein. Asn209 glycosylation was confirmed by a band shift visible on membrane stained with Coomassie Brilliant Blue (CBB). The full-length membrane is presented in Supplementary Figure S2. Contrast of the Coomassie-stained membrane was enhanced equally across the entire image. All bands remained visible after the digital processing. (**d**) Stability of IgMs with P1 position mutated into other 19 amino acids. Analyzed samples were derived from the same experiment but resolved in two different gels because of the limited number of wells. The figure presents images of two different blots processed in parallel. (**e**) Stability of chimeric mouse/human IgM. (**f**) Functional affinity of mutated IgMs analyzed by ELISA on immobilized human erythrocytes bearing a cognate antigen^7^. The results are representatives of three (**b** and **e**) or two (**c**,**d**,**f**) independent experiments. Bands corresponding to HC in blots presented in panels **c** and **d** are slightly overexposed in order to make μ’ signal more visible. Serial exposures of the overexposed blots are provided in Supplementary Figure S3.

Next, we aimed at elucidating the mechanism of truncation of the IgM heavy chain. We assumed that the cleavage is catalyzed by a serum protease^7^ and, to determine its specificity, we identified the residues within P6-P4′ sequence essential for trimming (Fig. 2a). Alanine screening revealed that among the investigated positions only Asn209 at P1 is crucial for this process (Fig. 2b).

In many antibodies asparagine is spontaneously deamidated into aspartic acid^14^. This process is relatively slow^15^ and progresses during protein storage^14^. We hypothesized that the truncation of the IgM heavy chain may follow deamidation of Asn209, which could be the rate-limiting step. Thus, we generated the Asn209Asp mutein and incubated it in neutral or acidic pH, in which the Asp residue is deprotonated or protonated, respectively. The mutein was resistant to fragmentation under both conditions (Fig. 2c). We also introduced an artificial N-glycosylation site on Asn209 by mutating Asp212 into Ser. The N-glycosylation on Asn209 was confirmed by a slight band shift to higher molecular mass in SDS-PAGE gel (Fig. 2c). The N-glycosylation of Asn209 also blocked IgM heavy chain cleavage (Fig. 2c).

Considering that many proteases are not able to hydrolyze a peptide bond formed by imine group of proline, we generated additional P1’ mutant Arg210Pro to verify whether a putative IgM-specific protease belongs to this category. However, despite many efforts, we were not able to efficiently express this IgM variant (Fig. 2c).

The results indicated that IgM truncation depends on the particular Asn residue, which provides the carboxyl group to the cleaved peptide bond. To confirm this observation we mutated Asn at P1 site into all other 19 protein amino acids and analyzed stability of generated muteins. The experiment proved that Asn209 is essential for IgM cleavage (Fig. 2d).

Despite the fact that in human IgMs Asn is present at the 209 position and is followed by a basic Lys residue, human IgMs are not susceptible to extracellular truncation in human serum (data not shown). To answer the question whether the sequence surrounding the scissile bond or other parts of the molecule are responsible for mouse IgM susceptibility to truncation, we generated a chimeric molecule: mouse IgM with the linker between CH1 and CH2 domains along with the preceding loop substituted for the human IgM-derived sequence. The obtained mouse/human IgM was resistant to fragmentation in the presence of serum (Fig. 2e), indicating that the features promoting cleavage are located within the replaced fragment (amino acids 203–239).

Trimming of IgM correlates with loss of IgM activity^7^. We believe that the obtained muteins resistant to the truncation of a heavy chain may address flaws of IgMs application in diagnostics or basic research^5–7^. Importantly, as presented in Fig. 2f, the introduced mutations did not affect affinity of IgM to the antigen.

We concluded from this part of the study that trimming of the mouse IgM heavy chain results from the exceptional sequence of the C-terminal fragment of its CH1. We also proved that cleavage could be prevented by mutating Asn209.

### Mouse IgM cleavage is a non-enzymatic process

We have previously reported that IgM truncation occurs in mouse serum^7^. Moreover, we traced FBS as a source of the cleaving factor in the case of IgMs secreted by hybridoma cells. The truncation was also observed in the presence of BSA obtained from bovine serum by heat fractionation. The BSA preparation contained numerous other proteins, thus we initially assumed that the cleaving factor co-purifies with BSA^7^.

High specificity of the cleavage pointed to an enzyme-mediated process. We searched MEROPS database^16^ for proteases specific to Asn at P1. Among few hits there was only one mammalian enzyme – legumain – a lysosomal cysteine protease^17^ that is also present in serum^18^. We hypothesized that legumain is the factor cleaving IgMs.

**Figure 3:**
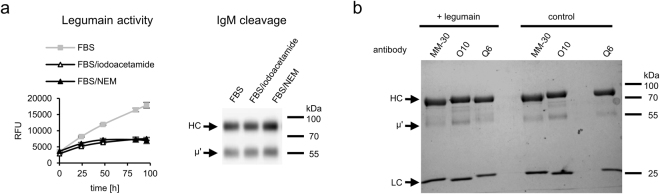
Legumain does not cleave mouse IgM. (**a**) IgM cleavage in serum treated with alkylating agents. Alkylating agents, such as iodoacetamide or NEM, are potent inhibitors of legumain – a cysteine protease specific to Asn at P1 site. Activity of legumain present in FBS was tested using fluorogenic substrate Z-Ala-Ala-Asn-AMC. Samples were analyzed using western blotting with anti-mouse IgMκ antibody. The full-length blot is presented in Supplementary Figure S2. (**b**) Recombinant legumain did not cleave IgMs. The gel was stained with Coomassie BB. (**a** and **b**) Representatives of two independent experiments are shown.

To verify this hypothesis, we inhibited legumain present in serum using alkylating agents (Fig. 3a) and then analyzed whether these reagents prevented IgM trimming. Moreover, we tried to cleave IgMs with active recombinant legumain (Fig. 3b). The results of these experiments were negative and indicated that legumain is not involved in IgM truncation.

**Figure 4:**
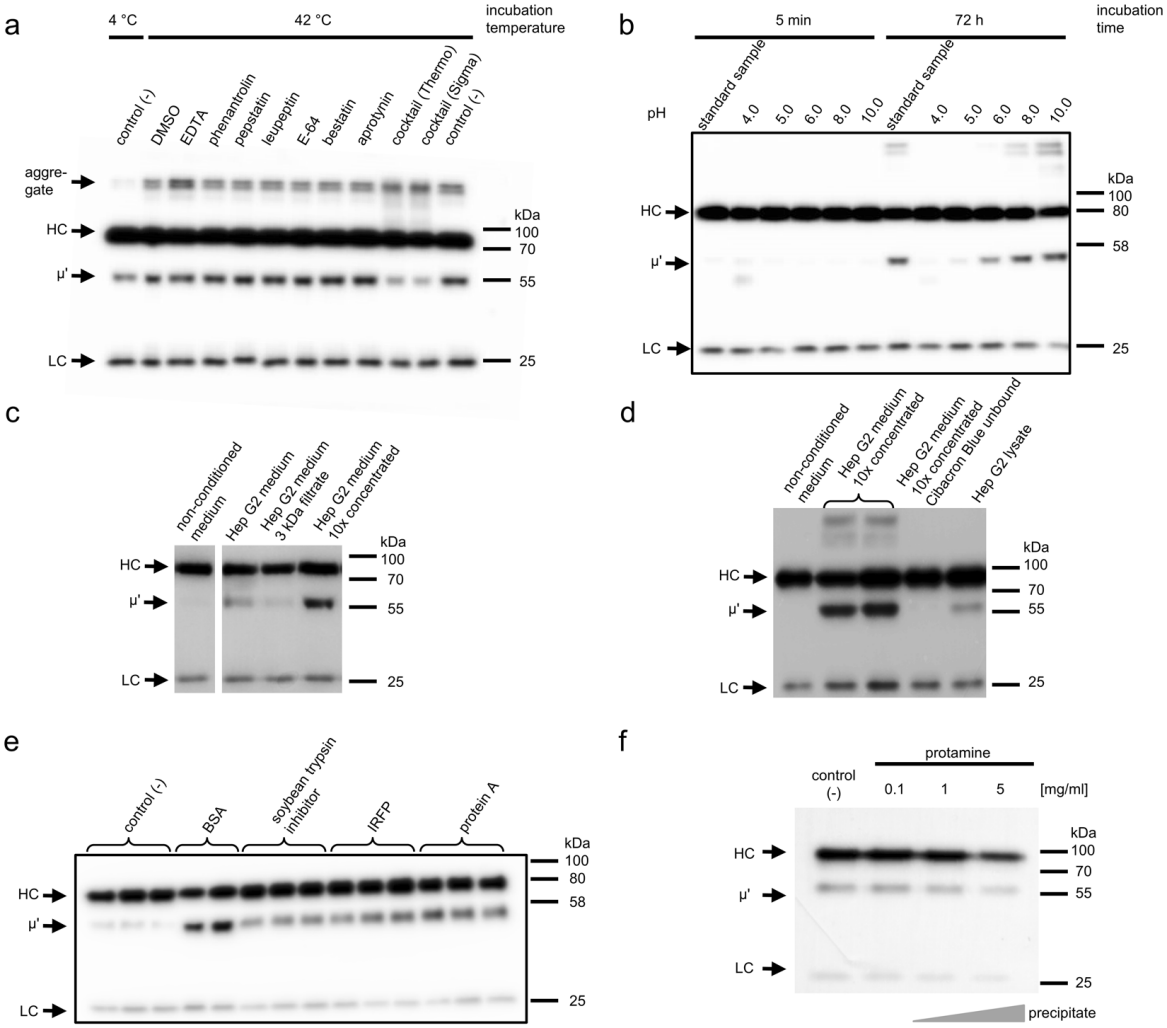
Factors affecting IgM cleavage. Samples were analyzed using western blotting with anti-mouse IgMκ antibody. (**a**) Serum-induced IgM cleavage in the presence of protease inhibitors. Presented result is an example of many experiments, in which also other inhibitors, all listed in Methods section, were used. None of the single inhibitors prevented IgM trimming. (**b**) Serum-induced IgM cleavage at different pH. Standard sample – medium without additional buffer. (**c** and **d**) IgM cleavage induced by serum-free medium collected from Hep G2 cell culture. The medium was concentrated by ultrafiltration with 3 kDa cut-off. Cibacron Blue resin removes a factor cleaving IgM from the medium (**d**). The image in panel **c** combines data extracted from one blot presented in full in Supplementary Figure S2. (**e**) IgM cleavage induced by various proteins. IgM was incubated with a hundredfold excess of the indicated proteins. Samples were analyzed in duplicates or triplicates. (**f**) IgM stability in the presence of protamine. Representatives of ten (**a**), two (**b**,**e**,**f**), or four (**c**,**d**) independent experiments are shown.

To identify the class of a putative protease involved in IgM cleavage we incubated the antibody with FBS (as a source of the enzyme) and a panel of protease inhibitors (Fig. 4a). We observed that protease inhibitor cocktail apparently limited IgM cleavage, but the inhibitors used as single agents were insufficient to block trimming. Although we tested 18 different inhibitors, which are listed in Methods section, we did not identify any specific inhibitor preventing IgM trimming. In our previous study we observed that protease inhibitor cocktail strongly enhances IgM aggregation^7^. Alike, in the present experiment the diminished accumulation of μ’ chain in samples containing the cocktail was accompanied by increased levels of aggregates and decreased levels of a full-length heavy chain (Fig. 4a). Therefore, we believe that low levels of μ’ chain in the cocktail-containing samples resulted either from the diminished cleavage due to IgM aggregation or from possible high propensity of μ’ chain to aggregate in the presence of the cocktail rather than from the specific action of particular inhibitors. Therefore, we decided to search for the cleaving factor *ab initio*.

Firstly, we analyzed the influence of pH on IgM cleavage (Fig. 4b). IgMs were trimmed more efficiently at neutral and alkaline pH than under acidic conditions. The result suggested that the cleaving factor cannot be a lysosomal protease.

Then, we analyzed whether the cleaving factor is secreted or is an intracellular protein occasionally released from dying cells. Most of the serum proteins are produced by hepatocytes. Thus we verified if the Hep G2 human hepatoma cell line secretes the cleaving factor. IgM trimming occurred during incubation of the antibody both in Hep G2 conditioned medium (Fig. 4c) and in Hep G2 cell lysate (Fig. 4d). Moreover, the cleaving factor could be concentrated by ultrafiltration using a 3 kDa cut-off membrane (Fig. 4c). The filtrate containing molecules with Mw lower than 3 kDa and negligible amount of proteins (∼20 μg/ml) did not induce IgM trimming, indicating that the cleaving factor is not a low Mw compound.

Following up on these results, we tried to fractionate the conditioned medium on Cibacron Blue resin, which binds many enzymes as well as serum albumin^19^. The results indicated that the resin retained the cleaving factor, because the flow-through fraction did not induce IgM truncation (Fig. 4d). The conditioned medium was also fractionated using glycoprotein-specific Concanavalin A resin. We observed that both the glycoprotein fraction and the flow-through fraction induced IgM truncation (data not shown). Although we searched for a specific factor involved in IgM trimming, the results suggested that there are different, secreted and intracellular, glycosylated and non-glycosylated, factors able to induce this phenomenon. This observation led us to the question on whether any protein could trigger μ’ generation.

To gain insight into IgM stability in the presence of other proteins we incubated IgM with an excess of BSA or other purified proteins that were readily available in our laboratory. The physiological serum level of albumin is about 20 mg/ml^20^. IgM concentration in non-immunized mouse serum reaches about 0.2 mg^21^ and increases after exposure to an antigen^7^. In our experiments we preserved the physiologic ratio between concentrations of albumin and IgM (100:1) and, for the experimental convenience, the total protein concentration was about 20 times lower than that in normal mouse serum.

This experiment revealed that IgM cleavage was induced, beyond BSA preparation, also by other acidic proteins: protein A, infrared fluorescent protein (IRFP) and soybean trypsin inhibitor (Fig. 4e).

We also incubated IgMs with three basic proteins: salmon protamine, hen egg lysozyme and mature vascular endothelial growth factor C (VEGF-C). In the case of protamine we observed precipitates, which quantity correlated with a concentration of protamine. Non-precipitated samples with the lowest protamine concentration (0.1 mg/ml) did not contain increased amount of truncated IgM (Fig. 4f). Lysozyme did not induce IgM trimming (data not shown). In samples with VEGF-C we did not observe precipitates, but IgM signals on exposed blots were substantially decreased (Supplementary Figure S1).

Presented results indicate that although extracellular truncation of mouse IgMs is a non-enzymatic process it depends on the presence of proteins with not yet fully defined characteristics. Our further studies aim to elucidate the mechanism of this cleavage.

## Discussion

Asn is a highly reactive residue that frequently contributes to inactivation and degradation of many proteins, including antibodies^22–24^. Asn is prone to non-enzymatic deamidation. Mechanism of the reaction is dependent on pH [reviewed in^23^]. Under acidic conditions, the side-chain amide bond is attacked by a water molecule, yielding ammonia and aspartic acid. At neutral or basic pH, two reactions are possible: (*i*) deamidation or (*ii*) cleavage. (*i*) The nitrogen in the peptide bond on the carboxyl side of Asn attacks the carbon in the amide group of Asn resulting in ammonia and a cyclic succinimide intermediate. The cyclic imide undergoes subsequent reactions involving isomerization or transpeptidation. (*ii*) Alternatively, the nitrogen in the amide group of Asn attacks the carbonyl in the following peptide bond. The reaction cleaves the protein backbone and yields two polypeptides, one of which ends with succinimide. The reactions (*i*) and (*ii*) compete at neutral or alkaline pH, but deamidation is favored over cleavage^25^ due to lower activation energy^26^. Deamidation at neutral or basic pH starts from deprotonation of the attacking nitrogen of the peptide bond. Cleavage occurs only if nitrogen deprotonation is hampered by e.g. Pro or a bulky side chain on the carboxyl side of Asn^27^. There is also a report suggesting that the protein tertiary structure may promote cleavage^26^.

Here we report non-enzymatic cleavage of the mouse IgM heavy chain after Asn209. Initially, we assumed that IgM truncation is catalyzed by a protease. However, we excluded involvement of legumain, the only known mammalian Asn (P1)-specific protease and we found that none of the inhibitors from the set that covered all protease classes was able to limit IgM trimming.

Observed dependence on neutral or alkaline pH suggests that IgM truncation may involve direct Asn-mediated cleavage or cyclic imide formation followed by other reaction(s) resulting in the peptide bond scission. The scissile bond in the IgM molecule is between Asn209 and Arg210. According to reports evaluating the influence of amino acids on the carboxyl side of Asn on its reactivity^28,29^, Arg210 may have two effects: a steric hindrance and base catalysis. Arg has a large side chain which may interfere with nitrogen deprotonation in a preceding peptide bond. A positive charge of Arg210 may also enhance nucleophilicity of Asn209 amide group, thus promoting the side-chain amid nitrogen attack on a carbonyl group in the peptide bond. Asn209 in folded IgM is surrounded by four basic residues: Lys201, His204, Lys208 and Arg210, which probably strongly influence its reactivity. Importance of basic amino acids in Asn-mediated peptide bond cleavage is described for inteins [reviewed in^30^]. Inteins are self-splicing proteins, which in their multi-step self-processing employ peptide bond cleavage after Asn by the mechanism described above as (*ii*). Inteins catalyze Asn-dependent scission through a unique three-dimensional structure preventing deamidation. The basic residues near Asn209 in IgM may promote cleavage in a similar way.

However, deamidation of Asn and Asn-dependent cleavage of a protein backbone are intramolecular processes, which do not require other proteins and do not depend on protein concentration^22^. Conversely, IgM truncation was observed only in the presence of other proteins. Purified IgMs even at a concentration of 250 μg/ml were not truncated unless incubated with serum, BSA^7^ or certain other proteins. We compared IgM trimming in the presence of BSA, IRFP, protein A and soybean trypsin inhibitor. Although all these proteins induced IgM cleavage, their efficacy was diverse. IgM trimming was induced most efficiently by BSA obtained from serum using heat treatment. The heat fractionation results in many impurities in the final preparation. Thus, we previously hypothesized that IgM cleavage is catalyzed by a protease co-precipitating with BSA^7^. However, the results obtained with IRFP, protein A and soybean trypsin inhibitor suggest that many non-enzymatic proteins may induce IgM trimming.

The analyzed proteins were acidic, with pI equal to or lower than 5.7 (5.7 – IRFP; 5.11 – protein A; 4.7 – BSA; 4.5 – soybean trypsin inhibitor). We also tried to use basic proteins: protamine (pI ~13), lysozyme (pI 11.4), and VEGF-C (pI 8.3). At high concentrations (>1 mg/ml) protamine precipitated during incubation with IgM, but at lower concentrations it neither influenced IgM quantity nor stimulated its trimming. Lysozyme also did not induce IgM trimming. In the presence of VEGF-C we observed a substantial loss of IgM. Because we did not observe precipitates in the samples with VEGF-C, we suppose that VEGF-C may enhance generation of IgM complexes unable to penetrate the gel.

What could be the mechanism of protein-induced IgM trimming? A tempting explanation is that a protein added to IgM solution may prevent Asn209 deamidation thereby shifting the balance towards cutting. Alternatively, the accompanying protein may induce a structural change in the IgM molecule which allows truncation. IgMs are complex proteins that form various types of oligomers^5,6^. According to the theory of molecular crowding [reviewed in^31^], the structure and oligomeric state of a protein may be modified by the presence of other proteins or crowding agents. In line with that, molecular crowding in serum modulated the fate of succinimide intermediate formed during Asn deamidation in a therapeutic antibody^24^. It is also possible that the added protein is directly involved in non-enzymatic peptide bond cleavage via a currently unknown mechanism.

Some authors consider Asn-dependent cleavage as a post-translational modification, which relates to protein maturation rather than degradation^32–36^. In the described examples the process was slow with a half-time ranging from about 200 to more than 10,000 days^15^. Thus, the Asn residue acted as a molecular clock enabling modification of a protein for a long time after its translation. Because of time requirements, Asn-dependent cleavage might be important in long-living polypeptides, e.g. proteins of the eye lens. At least two of them are subject to the Asn-dependent cleavage: crystallins^35^ and aquaporin-0^32^. This process modified their stability, packing and function and is believed to orchestrate lens development and participate in its ageing^33,34,36^.

The calculated half-life of mouse IgM in the circulation is about 2 days^37^. Despite the short life-time an evident amount of μ’ is constantly present in mouse sera^7,8^, indicating that truncation of IgM is faster than the known Asn-dependent peptide bond cleavage in other proteins. If the IgM trimming proceeds via already known reaction (*ii*), our results indicate that the process is accelerated by other proteins by a yet unrecognized mechanism. We cannot exclude that the phenomenon may be related to weak, non-specific catalytic capability of one or more serum proteins. Notably, serum albumins from a number of species are known to be able to catalyze certain reactions^38^. It is also possible, that IgM truncation occurs via a completely different reaction depending on labile Asn209.

A substantial amount of μ’ chain in mouse serum suggests that IgM truncation may be physiologically relevant. The identified site of heavy chain trimming is within CH1 domain, just before the linker joining CH1 with CH2. A cleavage at this site should produce a free antigen binding fragment (Fab) and a truncated heavy chain, which still remains within the oligomeric IgM. Both products may act as negative regulators of early immune response. The Fab fragment may bind to a cognate epitope and decrease antigen availability for other immunoglobulins. Truncated IgM has reduced avidity thus its interaction with an antigen is weakened. IgMs bound to multiple surface antigens e.g. on a bacterial surface are potent activators of the complement cascade, which leads to pathogen elimination. According to the current view, the complement activation requires a change in IgM conformation induced by antigen binding^39^. Binding sites for C1q, the first element of the complement cascade, are hidden in IgM structure and are exposed or formed only upon antigen binding^39^. IgM trimming resulting in removal of Fab may reduce IgM competence to activate complement. However, according to another model of complement activation by IgM^40^, spatial fluctuations of Fab fragments prevent C1q binding. Sequestration of Fabs by cognate antigen molecules allows interaction between C1q and IgM. Thus, a truncated IgM may be even more potent complement activator than the non-trimmed molecule, because of reduced requirements for the epitope availability.

Enzymatic cleavage of IgM is used by *Streptococcus suis* during host (swine) invasion to evade immune response^41,42^. The bacteria produce highly specific IgM protease Ide_Ssuis_, which cleaves porcine IgM heavy chain between CH2 and CH3 domains^41^. The action of Ide_Ssuis_ abrogated complement activation by porcine IgMs^41^. The products of IgM proteolysis induced by this pathogen differ from those of non-enzymatic IgM truncation in mouse, therefore the benefits or threats for mice, in which intrinsic IgM trimming occurs, are difficult to predict.

In conclusion, the mouse IgM heavy chain contains the labile Asn209 residue and is prone to non-enzymatic cleavage between CH1 and CH2 domains. The trimming occurs in neutral or alkaline pH and is induced by other proteins. The unique characteristic of this phenomenon suggests that it proceeds via currently unknown mechanism and may have pronounced physiological implications.

## Methods

### Antibodies and enzymes

To provide scientific integrity, we used three IgMs with different variable regions: O10 and Q6 IgMs were produced and purified as previously described^7^ and MM-30 was purchased from Abcam Cat# ab18401, Lot# G232282-2, G232282-3. We observed differences in proportion of full-length and truncated heavy chains in various IgM preparations. Despite our efforts to use non-trimmed antibodies only, some samples contained low levels of μ’ at the beginning of the experiment, because truncation of IgMs occurs already during their production by hybridoma cells. The antibodies (6–12 μg) were digested with PNGase F (Promega) under denaturing conditions according to the manufacturer’s instruction. Legumain (Novoprotein) was activated as described by Zhang *et al*.^43^. The activity of the enzyme was verified using the fluorogenic substrate Z-Ala-Ala-Asn-AMC (Bachem). The IgMs (10 μg) were incubated with activated legumain for 24 h at 37 °C and the substrate:enzyme mass ratio was 30:1. The incubation was performed in PBS and in the buffer suitable for legumain activity assays (20 mM citric acid, 60 mM Na_2_HPO_4_, 1 mM EDTA, 0.1% CHAPS, 1 mM DTT, pH 6.0). Samples of the IgMs treated with the enzymes were analyzed using SDS-PAGE and subsequent staining of the gels with silver nitrate or Coomassie Brilliant Blue.

### Cells

Hep G2 cells (ATCC #HB8065) were cultured in DMEM with 1 g/l glucose (Lonza) and 10% FBS (Biowest) at 37 °C, 5% CO2 in a humidified incubator. To prepare serum-free Hep G2 culture, the cells were cultured to reach 90% confluency and then washed 5× with PBS and 2× with DMEM. DMEM with 1 g/l glucose was added to the washed cells. Conditioned media were harvested after 24 h, centrifuged and filtered using 0.22 μm filters. HEK293 cells and hybridoma clones producing O10 and Q6 IgMs were cultured as described previously^7^. All cell cultures were free of *Mycoplasma* spp. and were routinely screened using PCR with *Mycoplasma* rDNA-specific GPO1 and MGSO primers^44^.

### Mass spectrometry

Bands were excised from SDS-PAGE gels and destained using 25% acetonitrile (ACN)/25 mM ammonium bicarbonate (ABC) and 50% ACN/25 mM ABC alternately. To reduce and alkylate proteins the samples were treated with 50 mM DTT/25 mM ABC for 45 min at 37 °C and then with 10 mg/ml iodoacetamide/25 mM ABC for 1 h at room temperature in the dark. Then, the bands were washed twice with 50% ACN/25 mM ABC, dehydrated in 100% ACN and air-dried. Gel pieces were re-swelled in 50 mM ABC containing 20 U of PNGase F (Promega) and incubated at 37 °C overnight. After that, they were extensively washed with ultrapure water, then with 50% ACN/25 mM ABC, dehydrated in 100% ACN and air dried. Gel pieces were re-swelled in 50 mM or 25 mM ABC containing Trypsin/Lys-C Mix (Promega) or V8 (Glu-C, Promega) enzyme and digestion was performed overnight at 37 °C. Then, trifluoroacetic acid (TFA) was added, peptides were collected, vacuum-dried and suspended in loading buffer (2% ACN, 0.05% TFA) for LC-MS/MS analysis done with Q Exactive mass spectrometer (Thermo Scientific) coupled with nano-HPLC (UltiMate 3000 RSLCnano System, Thermo Scientific) through a Digital PicoView 550 ion source (New Objective). Peptides were loaded onto trap column (AcclaimPepMap100 C18, Thermo Scientific; ID 75 μm, length 20 mm, particle size 3 μm, pore size 100 Å) in 2% ACN/0.05% TFA at a flow rate of 5 μl/min and then separated on analytical column (AcclaimPepMapRLSC C18, Thermo Scientific; ID 75 μm, length 150 mm, particle size 2 μm, pore size 100 Å) using a 90 min gradient of ACN from 2% to 40% in the presence of 0.05% formic acid at a flow rate of 300 nl/min. Top 8 method was used for MS measurement with full MS and MS/MS resolution of 70 000 and 35 000 respectively. To identify peptides, cRAP protein database containing O10 and Q6 sequences was searched by Mascot 2.5 (Matrix Science) via Proteome Discoverer 1.4 (Thermo Scientific).

### Edman sequencing

Determination of N-terminal amino acid sequence by Edman degradation was performed using PPSQ-31A automatic protein sequencer (Shimadzu). Before sequencing the proteins were separated on a SDS-PAGE gel, electrotransferred on a Immobilon P^SQ^ PVDF membrane (Millipore) and stained with Coomassie Brilliant Blue.

### Generation of plasmids coding for mutated mouse IgMs

To generate cDNA coding for a full-length IgM heavy chain, cDNA coding for the heavy chain variable region of mouse M18 antibody^7^ was cloned into pFUSEss-CHIg-mM plasmid (Invivogen) using *EcoR*I and *Nhe*I restriction enzymes resulting in pFUSEss-CHIg-mM_M18 plasmid. Q5-based site directed mutagenesis kit (New England Biolabs) was used to introduce mutations within the constant region of mouse IgM encoded in pFUSEss-CHIg-mM_M18. A plasmid coding for chimeric mouse/human IgM heavy chain was generated by replacing the sequence encoding the linker between CH1 and CH2 domains (amino acid residues 90–115 in the sequence provided by UniProtKB record P01872; IGHM_MOUSE) for the homologous human sequence (amino acid residues 90–115 in the sequence provided by UniProtKB record P01871; IGHM_HUMAN). A synthetic DNA coding for a fragment of mouse IgM with the human linker (Gene Art, Germany) was cloned into pFUSEss-CHIg-mM_M18 using endogenous restriction sites *Nco*I and *BamH*I. All generated plasmids were sequenced (Genomed, Poland) and are available via Addgene repository (plasmid numbers 91729–91756 and 91781) along with their complete sequences.

### Expression of recombinant IgMs and analyses of their cleavage

All recombinant IgMs were transiently expressed using adherent HEK293 cells cultured in DMEM supplemented with 10% FBS. The plasmids encoding IgM heavy chain (pFUSEss-CHIg-mM_M18 and its derivatives) were co-transfected with the plasmid coding for the cognate kappa light chain (pFUSEss-CLIg-mK_M18^7^) using Lipofectamine2000 (Invitrogen). Media collected from cultures of the transfected cells were centrifuged at 10,000 × g, buffered with 20 mM Tris-HCl pH 7.4 or 20 mM acetate buffer pH 4.0 and then incubated for 72 h at 42 °C. The samples were immunoprecipitated using CaptureSelect IgM Affinity Matrix (Thermo Fisher Scientific). For efficient binding to the matrix, pH of the samples incubated under acidic conditions was adjusted to 7.4 with 2 M Tris. The matrix (20 μl) with bound IgM was washed 4 times with 1 ml of PBS containing 0.05% Tween-20 and then boiled with SDS-PAGE reducing loading buffer. The samples were analyzed by western blotting using HRP-labeled goat anti-mouse IgMκ antibody (AbD Serotec, Cat# 5276–2504).

### Analyses of factors affecting IgM cleavage

Several factors affecting IgM cleavage were investigated. IgMs were incubated under conditions described below at 42 °C for 72–96 h and then boiled with SDS-PAGE loading buffer. The quantities of emerging μ’ chain were estimated by western blotting using goat anti-mouse IgMκ antibody.

(i) To inhibit activity of legumain, 50% FBS was treated overnight with 100 mM iodoacetamide (Sigma) in 250 mM carbonate buffer, pH 8.0. Alternatively, 50% FBS was incubated with 50 mM N-ethylmaleimide (Sigma) in 250 mM phosphate buffer, pH 6.5. FBS samples were then dialyzed three times against PBS. Each time the volume of dialysis buffer was fifty-fold the volume of sample. O10 or Q6 IgM (200ng) was incubated in 20 μl of PBS with 1.5 mg/ml treated or control FBS and then immunoprecipitated as described above. Legumain activity in the samples was verified using Z-Ala-Ala-Asn-AMC according to the protocol of the substrate’s manufacturer.
(ii) In studies with protease inhibitors, MM-30, O10, or Q6 IgM (200 ng) were incubated in 20 μl of PBS with 5% FBS (Biowest) and one of the inhibitors: AEBSF, antipain, aprotinin, benzamidine HCl, bestatin, chymostatin, E-64, EDTA, ε-aminocaproic acid, iodoacetamide, N-ethylmaleimide, leupeptin, pepstatin A, phosphoramidon, soybean trypsin inhibitor (all from Sigma), phenantrolin (Bioshop), batimastat (Sigma) or the legumain-specific inhibitor MV026630^45^, which was kindly gifted by Prof. Colin Watts (School of Life Science, University of Dundee, UK). Cocktails of inhibitors (Sigma or Thermo Scientific) were added to some samples in each experiment. The inhibitors were used in two concentrations: one recommended by their supplier and the second that was 2–10 times higher than the recommended one.
(iii) To analyze pH-dependency of IgM cleavage, 2.5 × 10^5^ hybridoma cells secreting O10 or Q6 IgM were seeded in 1 ml of DMEM with 5% FBS. The cultures were harvested after 24 h and centrifuged at 1000 × g for 10 min. The supernatants were then buffered with 20 mM citrate buffer pH 4.0 or 5.0; 20 mM phosphate buffer pH 6.0 or 8.0; or 20 mM Tris HCl pH 10.0. The buffered samples were then incubated for 5 min or 72 h.
(iv) Experiments with Hep G2 cell line were performed to establish localization of the cleaving factor. 200 ng of purified O10 IgM was incubated with 20 μl of: fresh serum-free medium (control) or tenfold concentrated serum-free Hep G2 culture medium, or Hep G2 cell lysate (10 μg of protein) obtained by mild sonication of cells suspended in PBS. The medium was concentrated by ultrafiltration using a 3 kDa cut-off membrane (Millipore). In some experiments the medium was pretreated with Cibacron Blue 3GA agarose (Sigma).
(v) Stability of IgMs in the presence of various proteins was also investigated. O10 or Q6 IgM (10 μg/ml) was incubated in 1 mg/ml solution of BSA obtained by heat-fractionation (Sigma) or highly purified IRFP (kindly provided by Jarosław Jucha from the Jagiellonian University in Kraków, Poland), lysozyme (Sigma), salmon protamine (Sigma), protein A (Thermo Scientific), soybean trypsin inhibitor (Sigma). VEGF-C was produced as previously described^46^ and used at a concentration of 200 μg/ml. Three different concentrations of protamine, 5, 1 and 0.1 mg/ml, were used. All solutions were prepared in PBS.

### SDS-PAGE and western blotting

The analyses were performed as previously described^7^. The images were analyzed using Fusion Fx apparatus with the Fusion Capt Advance Fx5 program (Vilbert Lourmat, France). Unless otherwise stated, the acquired images were not processed.

## Electronic supplementary material

Supplementary Figures
