# Supplementary material for: Mouse Antibody of IgM Class is Prone to Non-Enzymatic Cleavage between CH1 and CH2 Domains
Source: Sci Rep. 2018 Jan 11;8:519. doi: 10.1038/s41598-017-19003-4 (PMC5764968; doi:10.1038/s41598-017-19003-4)
Supplement: Supplementary file 1 — Supplementary Figures [file 41598_2017_19003_MOESM1_ESM.pdf]

Mouse Antibody of IgM Class Is Prone to Non-Enzymatic Cleavage between CH1 and CH2 Domains

Supplementary Figures

Tomasz Klaus[1,2], Krystyna Stalińska[2], Dominik Czaplicki[2], Paweł Mak[3], Bozena Skupien-Rabian[1,4], Sylwia Kedracka-Krok[4], Karolina Wiatrowska[2], Monika Bzowska[2], Monika Machula[2], Joanna Bereta[2]*

[1]Malopolska Centre of Biotechnology, Jagiellonian University in Kraków, Gronostajowa 7A, 30-387 Kraków, Poland; [2]Department of Cell Biochemistry, [3]Department of Analytical Biochemistry, [4]Department of Physical Biochemistry, Faculty of Biochemistry, Biophysics and Biotechnology, Jagiellonian University in Kraków, Gronostajowa 7, 30-387 Kraków, Poland;

* To whom correspondence should be addressed: Joanna Bereta, Department of Cell Biochemistry, Faculty of Biochemistry, Biophysics and Biotechnology, Jagiellonian University in Kraków, Gronostajowa 7, 30-387 Kraków, Poland; telephone: +48 12 664 63 56; Fax: +48 12 664 69 02; e-mail: joanna.bereta@uj.edu.pl

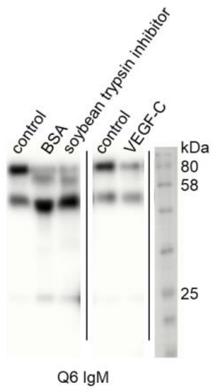

Supplementary Figure S1 – **IgM cleavage induced by various proteins.** IgMs were incubated at 42 °C for 72 h with a hundredfold excess of the indicated proteins. Then, the samples were analyzed using western blotting with anti-mouse IgMκ antibody. The image combines data from one blot. Representative result of two independent experiments is shown.

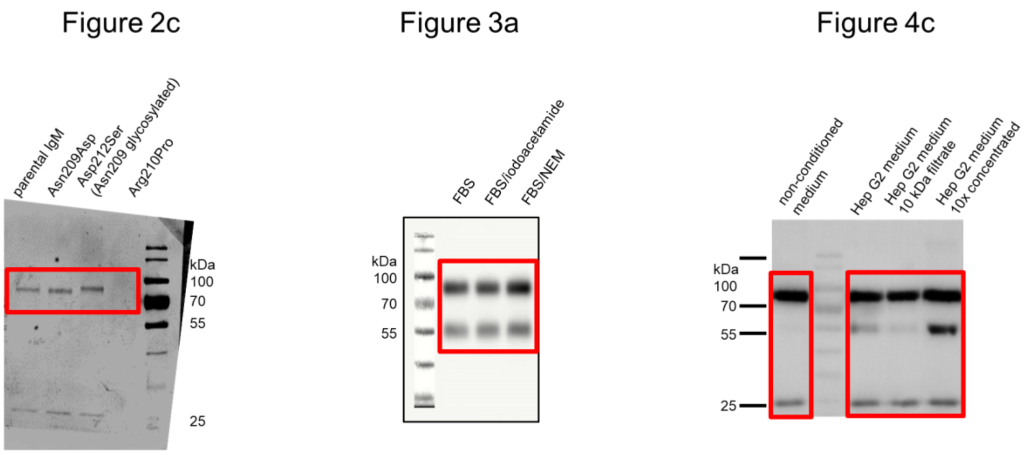

Supplementary Figure S2 – **Full-length versions of blots presented in the main manuscript.** Red frames indicate fragments presented in the Figures 2c, 3a and 4c in the main text.

Figure 2c

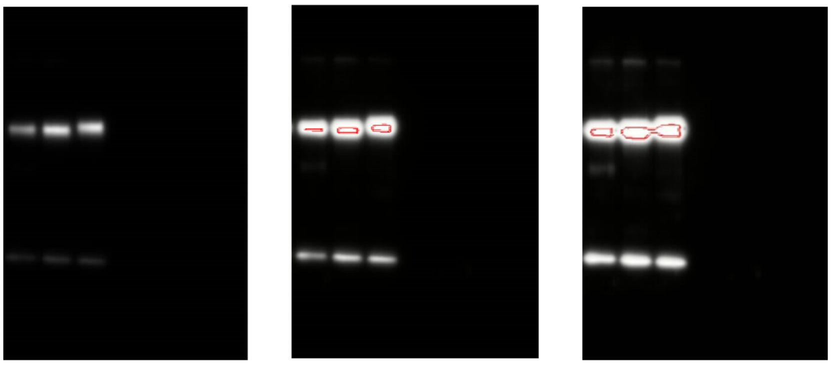

Figure 2d

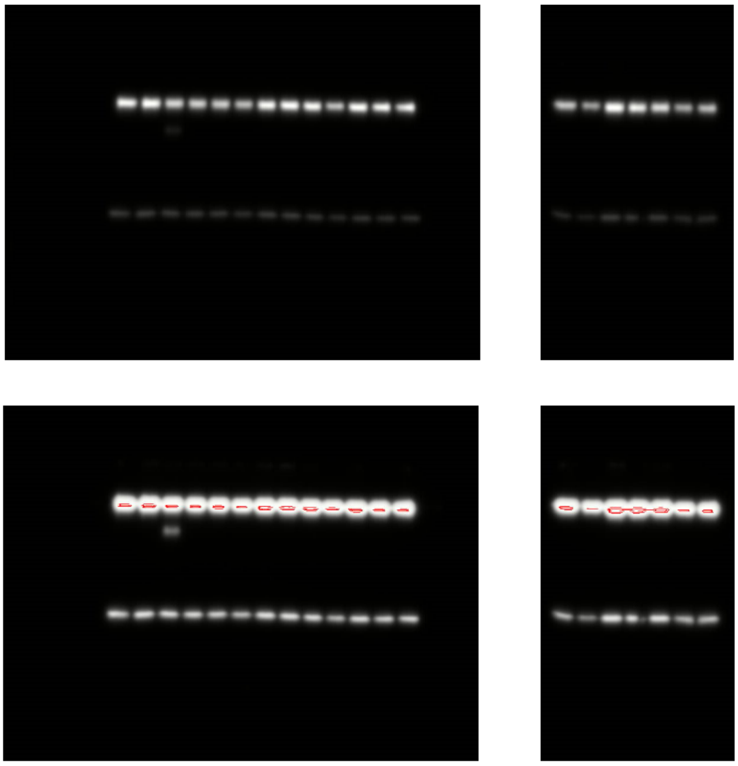

Supplementary Figure 3 – **Serial exposures of overexposed blots presented in Figures 2c and 2d in the manuscript.** Overexposed areas are marked in red.
